# The effectiveness of a group psycho-educational program on family caregiver burden of patients with mental disorders

**DOI:** 10.1186/1756-0500-5-399

**Published:** 2012-08-01

**Authors:** Ali Navidian, Fatihe Kermansaravi, Shahindokht Navabi Rigi

**Affiliations:** 1 Department of Mental Health & Psychiatric Nursing, Pregnancy Health Research Center, Zahedan University of Medical Sciences, Zahedan, Iran; 2 Department of Nursing, Pregnancy Health Research Center, Zahedan University of Medical Sciences, Zahedan, Iran; 3 Department of Midwifery, Pregnancy Health Research Center, Zahedan University of Medical Sciences, Zahedan, Iran

**Keywords:** Mental disorders, Caregivers, Education, Iran

## Abstract

**Background:**

Brief family intervention may have a positive impact on family caregivers for patients with mental disorders. We assessed the effectiveness of a group psycho-educational program on family caregivers for patients with schizophrenia and mood disorders.

**Methods:**

This randomized controlled trial was performed on 100 caregivers for patients with mental disorders attending the Isfahan Behavioral Sciences Research Center (IBSRC), in Isfahan, Iran. One hundred family caregivers of patients with schizophrenia (n = 50) and mood disorders (n = 50) were selected and assigned randomly to either a psycho-educational group intervention or routine care in each diagnosis category. The caregivers were followed for 3 months. Caregiver burden was assessed using the Zarit Burden Interview

**Results:**

The mean scores of the Zarit caregiver burden decreased significantly for the group that participated in the psycho-educational program, while scores in the control group did not change significantly.

**Conclusions:**

This group intervention program was effective to reduce the caregiver burden for both categories of mental disorders in the Iranian population. This group intervention program may improve the quality of life of patients and caregivers by improving the standards of care giving.

**Trial registration:**

RCT registration number: IRCT138804272200N

## Background

The importance of caregiver burden was pointed out as early as the 1950s [1]. Over the past two decades, the focus of the deinstitutionalization movement has shifted from psychiatric hospitals to community mental health centers. However, enough money has not been allocated towards such resources.

Family members of patients with seriously mental illnesses were encouraged to participate in this study to gain an understanding of practical help and emotional support for these patients [2,3]. They often provide significant services at home to their relatives who need their help; however, lack of sufficient information and resources has always been an obstacle. As a result, they are not well-prepared to play their roles as efficiently as possible [3-5].

Although care giving may have multiple rewards [6], extensive studies indicate that the intensity and diversity of care giving leads to caregiver strain and burden [7-9]. Family caregivers experience relationship strains, and feelings of grief, loss, sadness, anger, frustration, shame, and guilt as a consequence of caring for a relative at home [10]. Such strains and burden, if left untreated, can result in poor physical and mental health in the family caregivers [11,12]. As a consequence, this burden may reduce the quality of care giving and endanger the mental and physical health of the caregivers themselves [13]. Therefore, interventions such as education, support, psychotherapy, and respite, can be effective in reducing caregiver burden. These interventions enhance the quality of care giving, as well as the physical and mental health of caregivers [14,15]. Thus, reducing caregiver burdens provides better professional mental health outcomes for patients with mental disorders and their caregivers.

Although numerous studies have been performed over the past three decades, more studies need to enhance the development and evaluation of effective family intervention strategies. Psychiatric services for patients with severe mental illnesses and their families also need to be improved [16,17]. Group education programs have been widely recommended as a valuable strategy to deliver support and information to family caregivers [5].

Several other studies have also demonstrated the efficacy of family psycho-educational interventions in reducing of relapses, re-hospitalization [18-20] and family burden [21-23]. The psycho-educational intervention is a set of systematic interventions based on supportive and cognitive behavior therapy approaches with emphases on patients and family needs. The intervention is focused on increasing patient and family knowledge about mental disorders, adjusting to mental illness, and communicating and facilitating problem solving skills [24].

Iranian families are characterized by their intimate interpersonal relationships and many interactions among family members. Therefore, illness in one family member results in a substantial burden for the whole family. In addition, Iranian families report a low level of formal support services as compared with their Western peers [25]. Currently, there are no community mental health centers which specifically follow up on patients in Iran. The patients mainly are referred to psychiatrists, psychiatric centers, or primary healthcare centers that do not clearly address the specific needs of each family. Moreover, since mental illness is considered as a taboo in Iranian cultural settings, and many families are not aware of the needs and illness of their family members, they experience a great amount of burden. Also, neither the patients nor their families receive routine non pharmaceutical treatments such as family interventions. Finally, there are not trained professionals to perform such interventions in Iran.

Considering the lack of routine long term psychoeducational programs for patients and their families based on their specific needs, we investigated the efficacy of family psycho-education in reducing family caregiver burden.

## Methods

### Design

In a randomized controlled trial (RCT), we evaluated the effectiveness of a weekly, 4-session psycho-educational group intervention for caregivers of patients with mental disorders over a period of three months compared with routine care (control group) in the Isfahan Behavioral Sciences Research Center, in Isfahan, Iran. This study was done on a sample of family caregivers of patients with schizophrenia (n = 50) and mood disorders (n = 50). In each group, 25 caregivers were assigned randomly to either a psycho-educational group intervention or routine care.

In our study, a caregiver is defined as a member of the family who has the most frequent contact with the patient, financially supports the patient, has participated in the patient's treatment, is older than 15 years, and has good communicational skills. Family caregiver burden was assessed using the Zarit Burden Interview (ZBI) at baseline, at the end of the intervention period, and three months after the intervention. The results were compared between each group.

### Ethical considerations

Ethical approval was given by the study hospital’s ethics committee and the hospital’s research governance recommendations were followed. Written informed consent for participation in the study was obtained from family caregivers. An information leaflet about the study was distributed to family caregivers of patients with mental disorders.

This research was approved by the Ethics Committee of Isfahan Medical Sciences and was registered in the Iranian Registry of Clinical Trials (IRCT) with a reference number of IRCT138804272200N1.

### Instruments

Data were collected using the ZBI questionnaire. The ZBI is a widely used 22-item assessment tool for measuring the caregiver's perceived burden in providing family care. The questionnaire was translated, and modified according to Iranian cultural standards. Its reliability was calculated using the test-retest method (r =0.94).

The psychometric properties of the ZBI include an acceptable inter-item reliability and convergent validity, indicated by a Cronbach's alpha of 0.79 and a correlation coefficient of 0.71 between the caregiver's global evaluation and ZBI scores (Scott, Roberto, Hutton, & Slack, 1985). A test-retest reliability of 0.71 and internal consistency (Cronbach's alpha = 0.91) have also been reported (26–28).

This questionnaire asks family caregivers questions about physical, psychological, economic, and communication problems that cause stress and strain for caregivers. The items are answered on a five-point scale ranging from 0 (never) to 4 (always). Scores were calculated by summing up the total chosen statement which ranges from 0 to 88, that higher scores implying greater perceived caregiver burden.

### Intervention

We developed our intervention based on the families’ needs in schizophrenia and mood disorder groups and the existing literature (29, 30). The psycho-educational program consisted of four 120-min sessions held during four consecutive weeks with one session each week. Six psycho-educational groups of eight or nine caregivers (three groups for schizophrenia and three groups for mood disorders) were arranged with the same content, and the program was conducted by a mental health nurse or psychiatrist.

The goals and content of each of the four sessions are summarized in Table 1. At the beginning of the first session, a needs assessment was performed in which we asked the caregivers about the types of issues and problems they have with their patients, and what they would like to know about their patient’s condition so that we could organize the interventions appropriately. After evaluating the subjects’ needs assessments, each psycho-educational session included a variety of educational techniques designed to enhance the participant's learning and maintain their attention (for example; visual aids such as charts, film presentations and Microsoft PowerPoint slideshows).

**Table 1:** The contents of the psycho-educational program

**Session**	**Session goal**	**Content**
**Pre Session**	To orient caregivers to the program, to create a trusting relationship between the caregivers and instructors	· Overview of the program and introduction of the instructors and members to each other.
		· Discussion of the importance of orientation to patient behaviors and symptoms
		· Completion of Zarit Burden Interview (ZBI) questionnaire by participants.
		· Assessment of family needs.
**1**	To understand the disorder, its symptoms and treatments, and its effects on patients and families	· Case presentation. The instructor offers explanations of the symptoms and behaviors, and their effects on the family.
		· Discussion of the etiology and treatments
		· Question-answer and group discussion.
**2**	To recognize the effect of medications and compliance, and to orient caregivers to the warning signs of relapse and relapse prevention	· A review of the previous session.
		· Discussion of positive and negative effects of drugs and problems related to side effects.
		· Emphasis on the importance of drug compliance.
		· Discussion about the warning signs of relapse.
		· Explanation of the family role in relapse prevention.
		· Question-answer and group discussion.
**3**	To manage the patient’s symptoms, to gain skills in coping with the patient’s symptoms, and to understand effective ways to express emotion and improve communication skills.	· A review of the previous session.
		· Discussion of the importance of effective communication skills in the family and with the patients when they have symptoms.
		· Explanation of skills for coping with some of the patients’ symptoms, and cognitive and behavioral techniques for managing patients’ symptoms
		· Exploring intense emotions towards the patient.
		· Discussion of expressing emotion and the emotional environment in the family.
		· Discussion of how to cope with the patient’s negative emotions (for example, suicide behavior in mood disorder patients).
		· Question-answer and group discussion.
**4**	To orient caregivers to stress management and relaxation for the family	· A review of the previous session.
		· Introduction of the importance of stress management in the family.
		· Discussion of ways to reduce stress.
		· Practicing relaxation methods during the session.
		· Question-answer and group discussion.
		· Conclusion

The first part of each session consisted of a lecture given by a psychiatrist or mental health nurse, and the last part of each session (50 min) consisted of a question-and-answer and group discussion period. During this period, the caregivers described situations and incidents related to their family members and discussed alternative ways of coping with and resolving their difficulties with care giving. During these sessions, the caregivers were informed about the patients' disorder patterns, and psychosocial issues were discussed. Moreover, the caregivers were taught how to care for themselves, monitor their own feelings, and how to manage symptoms efficiently. Coping skills and problem solving strategies were also taught in a group format to be the most effective.

### Statistical analysis

The SPSS v. 15 was used for statistical analyses. At baseline, socio-demographic characteristics of the two groups were compared using the chi-square test. Between-group comparisons of the variables were performed with Student’s *t*-tests, and repeated measurement analyses of variance were used to determine whether the improvements in these variables changed over time.

## Results

The mean age of the caregivers was 43.03 ± 11.90 years. Of these caregivers, 47% were the patients' parents, 22% were the spouses, 20% were the siblings, and 11% were the children. The majority of the participants (92%) were married, and 56% were housewives. The patients' mean age was 34 ± 13.14 years, and most of them (58%) were male. Table 2 summarizes the patients' and their caregivers' additional socio-demographic and clinical data.

**Table 2:** Clinical and socio-demographic data of the patients and their caregivers

**Demographic and clinical features**		**Percentage of patients**	**Percentage of caregivers**
**Sex**	Male	58	30
	Female	42	70
**Age**	< 20 years	13	2
	21-35 years	48	28
	36 years	39	70
**Marital status**	Married	65	92
	Single	35	8
**Educational background**	No studies/illiterate	3	0
	Primary education	48	58
	Higher education or more	49	42
**Occupational status**	Housekeeper	30	56
	Employee	11	13
	Unemployed	30	6
	Business & similar jobs	24	19
	Other	5	6
**Diagnosis**	Schizophrenia	50	-
	Mood disorders	50	-

Comparisons of the baseline scores of the variables (FCB Score for Schizophrenia and FCB Score for Mood disorders) did not detect any significant differences between the two groups. The findings after completion of the psycho-educational program and the three month post-intervention scores indicated statistically significant reductions in the family burden scores as compared with the baseline scores (Table 3).

**Table 3:** Family caregiver burden at Time 0 (baseline), time 1 (post-intervention) and time 3 (three months post-intervention)

**Variables**	**Experimental group (n=25) Mean ± SD**	**Control group (n=25) Mean ± SD**		**P value**
FCB Score for Schizophrenia				
Time 0	57.28 ± 10.60	57.40 ± 11.59		> 0.05
Time 1	32.12 ± 8.52	47.08 ± 10.33		0.001*
Time 2	35.80 ± 7.48	44.52 ± 8.28		0.003*
Effect of time			14.10	0.01*
Effect of treatment			11.32	0.002*
Interaction of time and treatment			20.68	0.001*
FCB Score for Mood disorders				
Time 0	52.48 ± 9.02	49.04 ± 11.07		> 0.05
Time 1	25.44 ± 6.81	55.76 ± 11.77		0.0001*
Time 2	29.44 ± 7.29	52.88± 10.50		0.0001*
Effect of time			48.07	0.001*
Effect of treatment			71.99	0.001*
Interaction of time and treatment			61.23	0.001*

These results regarding the family caregivers to patients with mood disorders showed that the mean scores of burden in the control group was 49.04, 55.76, and 52.88 at baseline, after intervention, and 3 months after intervention, respectively. The mean scores in the experimental group were 52.48, 25.44, and 29.44, respectively. These results are statistically significantly different for the mean burden scores of caregivers for patients with mental disorders between the experimental and control groups (P = 0.001, F = 71.99).

Moreover, the interaction between group members and the level of burden for the three stages measured was also significant (P = 0.001, F = 61.23), or, in other words, the decrease of burden in the experimental group is significant compared with the control group. These data demonstrate that group interaction reduced caregiver burden in the experimental group, with the mean burden score being reduced considerably from 52.48 before the intervention to 25.44 after the intervention. This decrease remained low three months after intervention. The mean scores at time 0 (baseline) and time 2 (three month post-intervention) indicated that the experimental group had improved steadily in the family caregivers’ burden of schizophrenia (P < 0.001).

For family caregivers of schizophrenic patients, the mean burden score in the control group was 57.40, 47.08, and 44.52 at baseline, after intervention, and three months after the intervention, respectively (Table 3), compared with the mean burden scores of 57.28, 32.12, and 35.80, respectively, in the experimental group. Variance analysis with repeated measures demonstrated a significant difference in the mean burden score between the two groups (P = 0.002, F = 11.32), with the mean burden score in the experimental group being lower than the control group. Also, there is a significant correlation between group interaction and the level of burden for the three stages of measurement (P = 0.001, F = 20.68). In other words, the burden in the experimental group decreased significantly as compared with the control group. Intervention had a positive effect on reducing caregiver burden in such a way that the mean burden score decreased considerably from 57.28 at baseline to 32.12 after intervention and still remained low three months after intervention. The mean scores at time 0 (baseline) and time 2 (three month post-intervention) indicated that the experimental group improved steadily in the family caregivers’ burden of mood disorders (P < 0.001).

## Discussion

Most caregivers in our study were women who also were housewives. In Iranian households, girls or women are responsible for taking care of children, patients, elderly, and disabled people in the family as a part of their daily household chores [13]. Studies in western countries also have shown that women and girls were usually the main caregivers at home with most women being under 60 years of age. The primary caregivers for the elderly are usually their daughters or wives [27,28].

Burden is one of the most commonly used measures to evaluate the effect of intervention. The results of our study confirmed the hypothesis that psycho-educational family intervention has a significant effect on reducing the family caregiver burden when provided in a routine manner [32] for caregivers of family members with schizophrenia and mood disorders. A prospective study on the relationship between burden and coping in caregivers for patients with schizophrenia and mood disorders [33] showed that these caregivers face similar levels of burden and use similar types of coping methods to deal with it. Another psycho-educational group intervention for relatives of patients with bipolar disorders showed that the relative's insight about the disease improved and their burden reduced after one year of follow-up [34].

Magliano and colleagues (2006) reported that 40% of their patients and 45% of their relatives had a significant improvement in their social interactions during the intervention period. Other RCTs that were performed on Chinese [35] and Indian [36] family caregivers for patients with mental disorders confirmed that scores significantly improved after structured psycho-educational interventions compared with routine out-patient care.

We have demonstrated that intervention specifically developed for caregivers for patients with schizophrenia and mood disorder in Iran positively can influence burden. Compared with previous studies in which intervention was designed and performed for only a specific group of family caregivers [14,34-36], the intervention used in our study was designed to be effective for care givers for patients with schizophrenia and mood disorders, who comprise considerable numbers of the hospital inpatients in Iranian psychiatric wards.

### Limitations of this study

The current study has some limitations. Each family caregiver's personal experience and perception of care could have differed considerably in the experimental and control groups because of personal, familial, and economical differences. The sample size was relatively small, so larger studies are needed to confirm these results. The improvements in the family caregivers’ burden were confirmed for a relatively short follow-up period of three months, compared to other studies with a follow-up duration of one year [37]. Therefore, further studies are needed to confirm the long-term effects of this family psycho-educational intervention. Also more studies are recommended to apply different psycho-educational models, and family to family interventions, etc.

## Conclusions

Our group psycho-educational intervention improves the family caregiver burden. Our findings provide evidence that psycho-educational group intervention can be an effective family intervention for Iranian caregivers for family members with mood disorders and schizophrenia. Based on our findings, psycho-educational services for patients and their caregivers are helpful, beneficial and effective. The psycho-educational intervention used in our study is simple, feasible, and cheap, and could prevent the recurrence of severe mental disorders and long hospital admissions, without increasing mental burden or reducing the satisfaction of family care givers.

As intervention was effective in decreasing the burden of family caregivers for patients with schizophrenia and mood disorders, it is also likely that this program will be applicable to other psychiatric disorders. Future investigations should focus on obtaining more precise estimates of the contributions of the specific components of this program in reducing burden. The knowledge of how to support family caregivers for persons with mental disorders should be included in the education of healthcare professionals in general, and for the psychiatric team, in particular.

## Abbreviations

FCB: Family Caregiver Burden; IBSRC: Isfahan Behavioral Sciences Research Center; RCT: Randomized Controlled Trial; SD: Standard Deviation; ZBI: Zarit Burden Interview.

## Competing interest

The authors declare that they have no competing interests.

## Author Contributions

AN: Main investigator, creating the research question and research design, supervising the study, drafting the article and the guarantor.FK: Data analysis and critical revision of the article. SNR: managing group sessions. All authors read and approved the final manuscript.
